# Temporal structure of national vs. world-level taekwondo competitions: a time-motion analysis in taekwondo

**DOI:** 10.3389/fspor.2026.1897013

**Published:** 2026-07-22

**Authors:** Hongwei Yan, Yuqiao Zhu, Hu Chen, Linguo Chen, Wang Ren, Qing Guo, Zhenghe Guo, HuiMeng Dong, Junli Yang, Qinjian Xu, Taining An

**Affiliations:** 1 Sport Coaching College, Beijing Sport University, Beijing, China; 2 China Sport and Health Research Institute, Beijing Sport University, Beijing, China

**Keywords:** competition intensity, effort-to-pause ratio, performance analysis, taekwondo, time motion analysis

## Abstract

**Purpose:**

International taekwondo competition has undergone substantial rule-driven intensification in recent years, yet empirical evidence regarding how Chinese athletes temporally adapt to these demands relative to global elites remains scarce. This study compared the temporal structure of the 2025 Chinese National Games (CNG) and 2025 Wuxi World Taekwondo Championships (WWC) to quantify differences in High intensity activities(HIA), Low intenisty activities(LIA), Referee pause(RP), and Effort: Pause(E:P) ratios.

**Methods:**

Time-motion analysis was used on 171 rounds (75 matches) of gold-medal bouts. A 41-indicator taxonomy was developed via the Delphi method and applied frame-by-frame by two certified referees (Cohen's *κ* = 0.86). Variables were compared using Kruskal–Wallis, Mann–Whitney U, and three-way aligned rank transform (ART) ANOVA tests.

**Results:**

No between-tournament difference was found in round duration (*p* > .05). However, WWC demonstrated longer HIA duration (*Z* = −7.046, *p* < .001, *r* = .54) and shorter LIA duration (*p* = .050, *r* = .15) than CNG. Specifically, WWC showed longer directional-change kicks ≥2, consecutive slides ≥2, and forceful clinching (all *p* < .01), but shorter body rotation ≥360° (*p* = .004, *d* = 0.49). For LIA, WWC exhibited shorter single kicks, and bouncing steps, yet longer static standing and single slides (all *p* < .01). Referee pauses were shorter in WWC for regular, penalty, and video-review stoppages (all *p* < .001). The E:P ratio was 1:3.49 at WWC vs. 1:4.17 at CNG; the HIA:LIA ratio was 1:1.56 vs. 1:2.18.

**Conclusions:**

The discrepancy between domestic and international elite taekwondo lies in intensity distribution rather than total round duration. Chinese domestic competition exhibits lower temporal density. Training should extend high-intensity bout duration, compress recovery, emphasise multi-directional combinations, prioritise clinch-breaking and close-quarters tactics, and develop explosive kicking from static to dynamic states.

## 1 Introduction

Taekwondo has evolved considerably since its Olympic debut at Sydney 2000. Successive rule revisions and the universal adoption of electronic protector and scoring systems (PSS) from London 2012 onwards have fundamentally altered the technical-tactical and physiological landscape of elite competition (1–3). The instantaneous feedback of electronic headgear has increased kicking frequency and accelerated attack-to-defence transitions (4, 5), while standardisation of an 8 m × 8 m contest area (2010) (6), progressively stricter penalties for passivity (introduction of direct Gam-jeom penalty points alongside the existing warning system, 2009) (7), and augmented scoring for turning kicks (trunk: 2 → 4 points; head: 3 → 5 points, 2009–2018) (8) have collectively elevated competitive intensity (2). Against this backdrop, the Chinese taekwondo team—historically dominant from 2000 to 2016 with consecutive Olympic gold medals (9)—experienced a marked decline in podium finishes at both Tokyo 2020 and Paris 2024 (9). While this downturn likely reflects multiple interlocking factors—including technical adaptation (4, 10), opponent evolution (11), and training methodologies (4, 12)—one underexplored dimension is whether the temporal structure of domestic Chinese competitions differs from that of world-level events, which simultaneously represent a potential contributing factor.

Competitive intensity in taekwondo is inherently intermittent, characterised by irregular bursts of high-intensity activity (HIA) interspersed with low-intensity activity (LIA) and referee-imposed pauses (RP) (13, 14). Time-motion analysis (TMA) offers a non-invasive, ecologically valid framework for quantifying these discrete activity patterns, thereby illuminating the technical, tactical, and physiological requirements of competition (15). Previous TMA studies in taekwondo have profiled activity distributions across weight categories (13, 16–20), sexes (16, 17, 19), competition rounds (13, 19, 20), and tournament levels (20), revealing that elite athletes spend approximately 30%–45% of round time in HIA, with effort-to-pause (E:P) ratios typically ranging between 1:2 and 1:8 (19–25). However, these investigations have largely been confined to single-event descriptions or cross-sectional comparisons of athlete subgroups within a single tournament; direct, systematic comparisons between national-level and world-level competitions remain scarce. Del Vecchio et al. (20), in the most closely related predecessor study, compared competitive levels within a single tournament (regional vs. state-level Brazilian championships) and reported longer HIA and superior E:P ratios at the higher competitive level. While this study established that competitive level modulates temporal structure within a single event context, it did not address whether temporal structure differences persist when comparing distinct tournament systems (e.g., national vs. world championships), nor whether such differences, if observed, might have implications for athletes transitioning between competition systems.

To the best of our knowledge, no published study has yet conducted TMA in taekwondo under the most recent rule cycle [post-2022 modification reducing the non-engagement penalty window from 10 s to 6 s (26)]. This rule change has plausibly intensified global competitive density by compelling more rapid attack initiation; however, whether national and international tournament systems have adapted equivalently to this intensification remains unknown. Understanding whether domestic Chinese competition replicates the temporal density of world-level competition bouts is relevant for training prescription. If domestic contests afford longer recovery windows, simpler technical exchanges, or more protracted referee stoppages, Chinese athletes may encounter a temporal structure discrepancy when transitioning to international arenas—though it must be emphasised that such a discrepancy, if identified, would represent a correlation rather than a causal explanation for international performance outcomes. The present study adopts a cross-sectional comparative design and therefore cannot adjudicate directionality, exclude confounding variables, or establish causal mechanisms linking temporal structure differences to competitive outcomes.

Accordingly, the present study aimed to: (i) compare round-level temporal variables between the 2025 Chinese National Games (CNG) and the 2025 Wuxi World Championships (WWC); (ii) contrast activity-level temporal structures (HIA, LIA, RP durations and frequencies) between tournaments; and (iii) quantify E:P and HIA:LIA ratios to evaluate relative competitive intensity. We hypothesised that WWC bouts would display a more compressed temporal structure—specifically, longer HIA, shorter LIA, and shorter RP durations—despite no difference in absolute round duration. We hypothesised that WWC bouts would display a more compressed temporal structure—specifically, longer HIA, shorter LIA, and shorter RP durations—despite no difference in absolute round duration, while acknowledging that any observed differences may reflect multiple factors including, but not limited to, athlete preparation, officiating conventions, and tournament-specific rules.

## 2 Materials and methods

### 2.1 Design and sample

This was a comparative observational study employing TMA. Video footage of all gold-medal bouts from the 2025 CNG (32 matches, 75 rounds) and the 2025 WWC (43 matches, 96 rounds) was retrieved from official broadcasts (Yangshipin and World Taekwondo). One round from the CNG was excluded due to incomplete footage, yielding a final sample of 171 rounds (75 matches). Only bouts captured from multi-angle, high-definition broadcasts were retained.

To ensure weight-category consistency, the study focused on the eight Olympic weight classes contested at both tournaments. Because the World Championships did not include the +67 kg (women) and +80 kg (men) divisions, the corresponding +73 kg and +87 kg categories were used as as the nearest available weight categories. While the Chinese athletes in these divisions were identical across both events, the international athlete composition in these substituted categories may differ from what would be expected in +67 kg and +80 kg. This substitution represents an ecological constraint rather than a true proxy.

### 2.2 Instrumentation and taxonomy

A 41-indicator temporal taxonomy was developed through a three-round Delphi consultation with seven experts (professors and national-team coaches; >10 years taekwondo-specific experience) and pilot-tested with four active elite athletes. The final system classified each frame into one of three mutually exclusive categories:

**HIA:** rapid displacement, attack, defence, or counterattack movements (e.g., same-leg consecutive kicks ≥ 2, clinch breaking, forceful clinching, body rotation ≥ 360°, jumping kicks). As shown in Supplementary Material.
**LIA:** static or low-velocity preparatory and recovery movements (e.g., single kick, bouncing step, static standing, single slide, passive clinching). As shown in Supplementary Material.
**RP:** referee-initiated stoppages (regular, penalty, video review, injury, equipment malfunction, unexpected incident). As shown in Supplementary Material.

Activity onset was defined as the frame in which a limb left the ground or an attack was initiated; offset was defined as the frame in which the limb returned to guard, the athlete fell, or the referee signalled “Kalyeo”.

### 2.3 Video analysis

Two researchers (both national first-class taekwondo referees) coded the footage using Kinovea 2023.1.2 at 0.05 s precision. Prior to formal coding, a two-week calibration protocol was completed using 10 non-sample matches. Inter-observer reliability was assessed on 20 randomly selected matches (Cohen's *κ* = 0.86), and intra-observer reliability was verified via repeated coding after a one-week washout.

### 2.4 Statistical analysis

Data were managed in Microsoft Excel 2021 (Microsoft Corp., Redmond, WA, USA) and analysed in IBM SPSS Statistics 27.0 (IBM Corp., Armonk, NY, USA), with “*α*” set *a priori* at .05. Normality was assessed using Kolmogorov–Smirnov (*n* > 50) or Shapiro–Wilk (*n* < 50) tests; homogeneity of variance was checked with Levene's test. Because most micro-temporal variables violated normality, non-parametric tests were preferred: Mann–Whitney U for two-group comparisons and a three-way aligned rank transform (ART) ANOVA for round × tournament × weight class interactions. Independent-samples t-tests were used only for normally distributed variables. Prior to ART ANOVA, cell-size balance was examined across all Tournament × Round × Weight Class combinations. Cell sizes ranged from *n* = 2 to *n* = 6 (max/min ratio = 3), indicating an approximately balanced design. Effect sizes are reported as rank-biserial correlation (r) for non-parametric tests and partial eta-squared (*η*^2^p) for ART ANOVA tests, interpreted using the following thresholds: trivial (<0.01), small (0.01–0.06), moderate (0.06–0.14), and large (≥0.14) (27). To control for type-I error arising from multiple comparisons, the Benjamini-Hochberg false-discovery-rate (FDR) correction was applied to adjust raw *P*-values, with *q*-value < 0.05 defined as statistically significant. *post-hoc* power analyses were performed using G*Power 3.1(two-tailed *Z*-test, ɑ = .05). Descriptive data are presented as mean ± standard deviation (M ± SD).

## 3 Results

### 3.1 Macro-temporal structure

**Figure 1:**
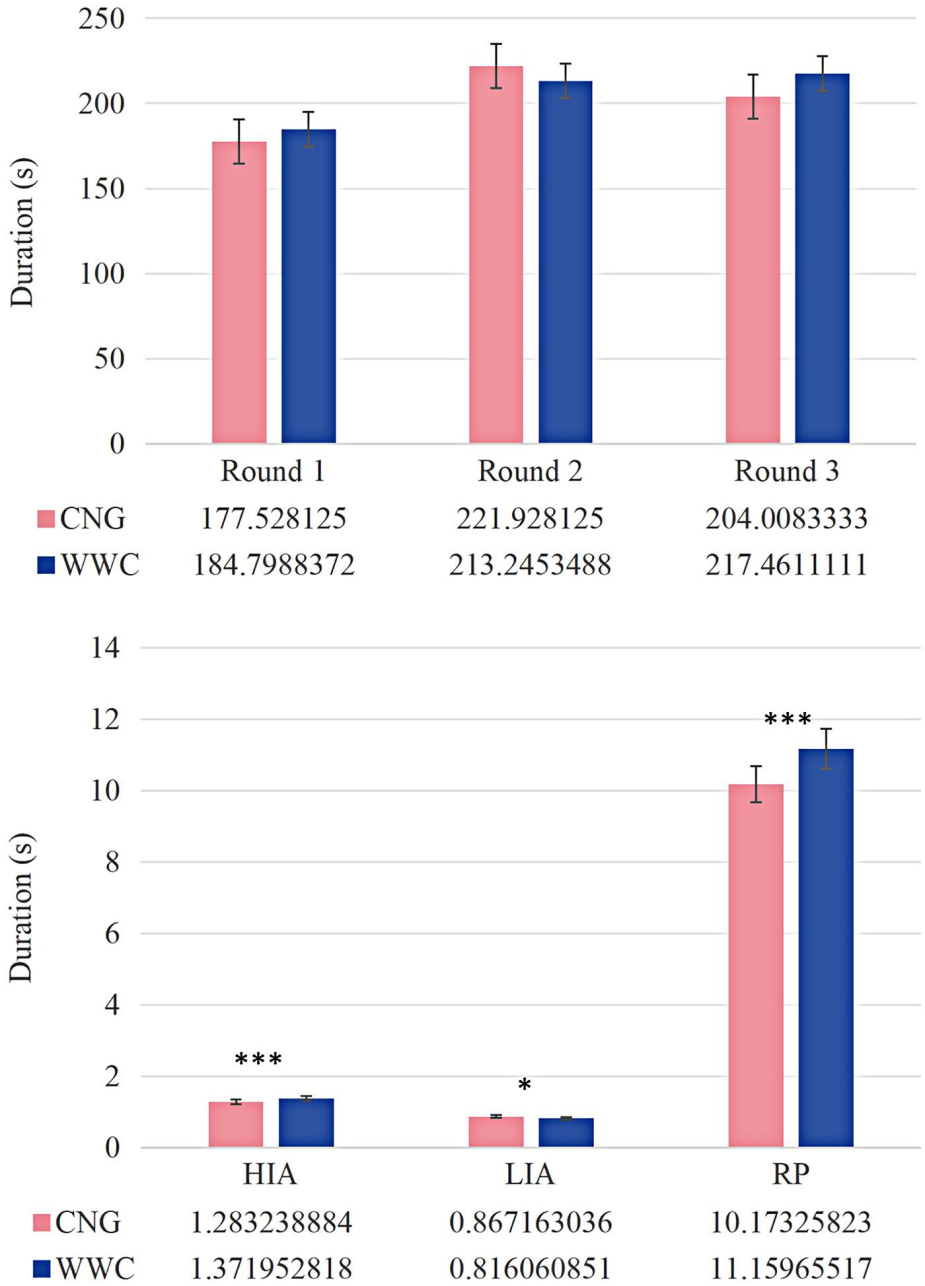
Round duration and aggregate durations of high-intensity activity (HIA), low-intensity activity (LIA), and referee pause (RP) by tournament.

Shapiro–Wilk tests indicated that round durations violated normality in the majority of tournament-round combinations (all *p* < .05 except WWC Round 3, *W* = 0.934, *p* = .104). Consequently, three-way aligned rank transform (ART) ANOVA (Tournament × Round × Weight Class) was conducted to examine round duration (Table 1, Figure 1).

**Table 1:** Three-way aligned rank transform (ART) ANOVA results for competition duration.

Source	*df*	*F*	*p*	*η* ^2^
Tournament (T)	1	.491	.485	.004
Weight Class (W)	7	1.297	.257	.068
Round (R)	2	3.408	.036*	.052
T × W	7	.660	.706	.036
T × R	2	.497	.610	.008
R × W	14	1.506	.118	.144
T × R × W	12	.603	.836	.055
Residual	125	—	—	—

* *p* < .05.

ART ANOVA revealed a significant main effect for round [F(2, 125) = 3.408, *p* = .036, *η*^2^p = .052, small], whereas the main effects for tournament [F(1, 125) = .491, *p* = .485, *η*^2^p = .004, trivial] and weight class [F(7, 125) = 1.297, *p* = .257, *η*^2^p = .068, small] were non-significant. None of the interactions reached significance (Tournament × Round: F[2, 125] = .497, *p* = .610, *η*^2^p = .008, trivial; Tournament × Weight Class: F[7, 125] = .660, *p* = .706, *η*^2^p = .036, small; Round × Weight Class: F[14, 125] = 1.506, *p* = .118, *η*^2^p = .144, moderate; Tournament × Round × Weight Class: F[12, 125] = .603, *p* = .836, *η*^2^p = .055, small). Although the main effect of round was significant, *post-hoc* pairwise comparisons did not reveal any significant between-round differences.

### 3.2 Micro-temporal structure

#### 3.2.1 Aggregate activity profiles

Kolmogorov–Smirnov tests confirmed that aggregate high-intensity activity (HIA), low-intensity activity (LIA), and referee pause (RP) durations all violated normality in both tournaments (all *p* < .001). Mann–Whitney U tests were therefore employed.

Mann–Whitney U tests revealed significant between-tournament differences for HIA, LIA, and RP durations (Table 2, Figure 1). WWC displayed longer HIA (*Z* = −7.046, *p* < .001, *r* = .54, large), shorter LIA (*Z* = −1.958, *p* = .050, *r* = .15, small, *post-hoc* power of 0.999), and shorter RP (*Z* = −5.291, *p* < .001, *r* = .40, moderate) than CNG.

**Table 2:** High-intensity, low-intenisty and referee pause activity durations (mean ± SD, s) by tournament.

	Activity	CNG		WWC		*Z*/*t*	*p*	Median difference (95% CI)	*q*
		*n*	Duration (*s*)	*n*	Duration (*s*)				
HIA	SCK	298	1.89 ± 0.73	393	1.89 ± 0.61	−.657	.511	−.050 (−.100,.050)	—
	ALK	85	1.63 ± 0.48	64	1.67 ± 0.50	−.626	.531	−.050 (−.200,.100)	—
	DCK*	147	1.30 ± 0.41	214	1.59 ± 0.45	−7.111	<.001	−.300 (−.350, −.200)	<.001
	CTK	5^a^	1.81 ± 0.43	2	1.25 ± 0.00	−1.972	.049 (.095^b^)	.500 (.100, 1.000)	—
	CSL*	643	1.23 ± 0.60	536	1.58 ± 0.41	−14.794	<.001	−.450 (−.500, −.400)	<.001
	PKC	5^a^	1.46 ± 0.48	9^a^	1.84 ± 0.68	−1.113	.287	−.400 (−1.100,.450)	—
	BKC	17^a^	1.85 ± 0.64	7^a^	1.81 ± 0.32	.139	.891	.000 (−.500,.450)	—
	CLB	339	0.99 ± 0.40	695	1.13 ± 0.43	−1.820	.069	−.150 (−.150, −.100)	—
	EBM	129	0.79 ± 0.33	124^a^	0.69 ± 0.22	−1.863	.062	.050 (.000,.100)	—
	FEA*	228	0.68 ± 0.23	319	0.65 ± 0.19	−2.859	.008	.050 (.000,.050)	.068
	CFM	201	2.06 ± 0.64	233	2.16 ± 0.77	−.652	.514	−.050 (−.150,.100)	—
	BFR*	13^a^	0.78 ± 0.21	16^a^	0.60 ± 0.11	3.103	.004	.150 (.050,.300)	<.001
	CDR	2	1.73 ± 0.39	1	3.00 ± 0.00	−1.225	.221 (.667^b^)	−1.275 (−1.550, −1.000)	—
	JPK	45	0.91 ± 0.29	49	0.88 ± 0.25	−.197	.844	.000 (−.100,.100)	—
	FCL*	137	1.08 ± 0.48	390	1.23 ± 0.49	−3.066	.002	−.150 (−.200, −.050)	.003
Total/Average		2,294	1.28 ± 0.68	3,052	1.37 ± 0.65	−7.046	<.001	−.100 (−.150, −.100)	<.001
LIA	SIK*	1,026	0.92 ± 0.52	897	0.91 ± 0.21	−2.794	.005	−.050 (−.050,.000)	<.001
	SGS*	758	0.47 ± 0.42	707	0.50 ± 0.16	−5.584	<.001	−.050 (−.050, −.050)	<.001
	SDA	1,463	1.10 ± 1.41	2,149	0.96 ± 0.50	−1.638	.101	−.050 (−.050,.000)	—
	SRA	410	1.08 ± 0.62	611	1.07 ± 0.47	−1.205	.228	−.050 (−.100,.000)	—
	BOS*	1,538	0.97 ± 0.63	1,052	0.79 ± 0.39	−5.583	<.001	.100 (.050,.100)	<.001
	SST*	793	0.77 ± 0.63	1,370	0.80 ± 0.53	−4.377	<.001	−.050 (−.100, −.050)	.008
	ARS	262	0.50 ± 0.20	248	0.50 ± 0.18	−.714	.844	.000 (−.050,.000)	—
	SBK	63	0.82 ± 0.22	44^a^	0.83 ± 0.19	−.197	.226	.000 (−.100,.050)	—
	SPK	26^a^	0.80 ± 0.24	27	0.80 ± 0.16	−.125	.900	.000 (−.100,.100)	—
	STP	3	0.65 ± 0.17	38	0.55 ± 0.22	−1.005	.315 (.345^b^)	.100 (−.150,.350)	—
	STB	81	0.43 ± 0.18	94	0.46 ± 0.18	−1.004	.315	−.050 (−.100,.050)	—
	JGB	92	0.55 ± 0.15	145	0.56 ± 0.19	−.088	.930	.000 (−.050,.050)	—
	MVB	183	0.57 ± 0.23	104	0.57 ± 0.17	−.811	.417	.000 (−.050,.050)	—
	STE	44	0.55 ± 0.26	31^a^	0.54 ± 0.17	−.557	.578	.000 (−.100,.050)	—
	GBP	2	0.78 ± 0.18	3^a^	1.10 ± 0.41	−1.155	.248 (.400^b^)	−.225 (−.900,.150)	—
	BPR	11	0.56 ± 0.27	15	0.46 ± 0.09	−.927	.354 (.384^b^)	.050 (−.050,.150)	—
	STK	23^a^	0.91 ± 0.26	25^a^	0.90 ± 0.19	−.243	.808	.000 (−.100,.150)	—
	PCL	374	1.00 ± 0.34	218	0.80 ± 0.29	.228	.821	.200 (.150,.250)	—
	SSW*	77	0.49 ± 0.14	67	0.46 ± 0.13	−2.017	.007	.050 (.000,.050)	.053
	LLI	162	0.59 ± 0.26	124	0.62 ± 0.55	−1.632	.103	.000 (−.050,.050)	—
Total/Average		7,391	0.87 ± 0.81	7,428	0.82 ± 0.45	−1.958	<.050	.000 (.000,.000)	<.001
RP	RPA*	435	5.02 ± 2.80	471	3.45 ± 1.47	−10.105	<.001	1.000 (.800, 1.250)	<.001
	PPA*	117	9.75 ± 3.42	165	7.48 ± 2.88	−6.412	<.001	2.150 (1.550, 2.800)	<.001
	VPA*	19	120.56 ± 77.93	71	66.93 ± 32.61	−4.187	<.001	42.550 (28.150, 61.650)	<.001
	IPA	4^a^	49.59 ± 31.47	5	35.37 ± 50.98	−1.225	.221 (.286^b^)	24.875 (−89.950, 68.700)	—
	EPA	1	39.15 ± 0.00	—	—	—	—	—	—
	UPA	1	14.90 ± 0.00	13	23.42 ± 19.58	−.372	.710 (.857^b^)	−4.900 (−59.300, 13.050)	—
Total/Average		577	10.17 ± 25.25	725	11.16 ± 21.92	−5.291	<.001	.750 (.450, 1.050)	<.001

* *p* < .05 vs. counterpart tournament.

a Normally distributed.

b Small-sample exact test.

#### 3.2.2 High-intensity activities

Shapiro–Wilk tests indicated that only three of the 15 HIA indicators satisfied normality assumptions in both tournaments: push-and-kick combinations ≥2, block-and-kick combinations ≥2, and body rotation ≥360° (all *p* > .05). These three indicators were compared using independent-samples t-tests; the remaining 12 were analysed with Mann–Whitney U tests.

**Figure 2:**
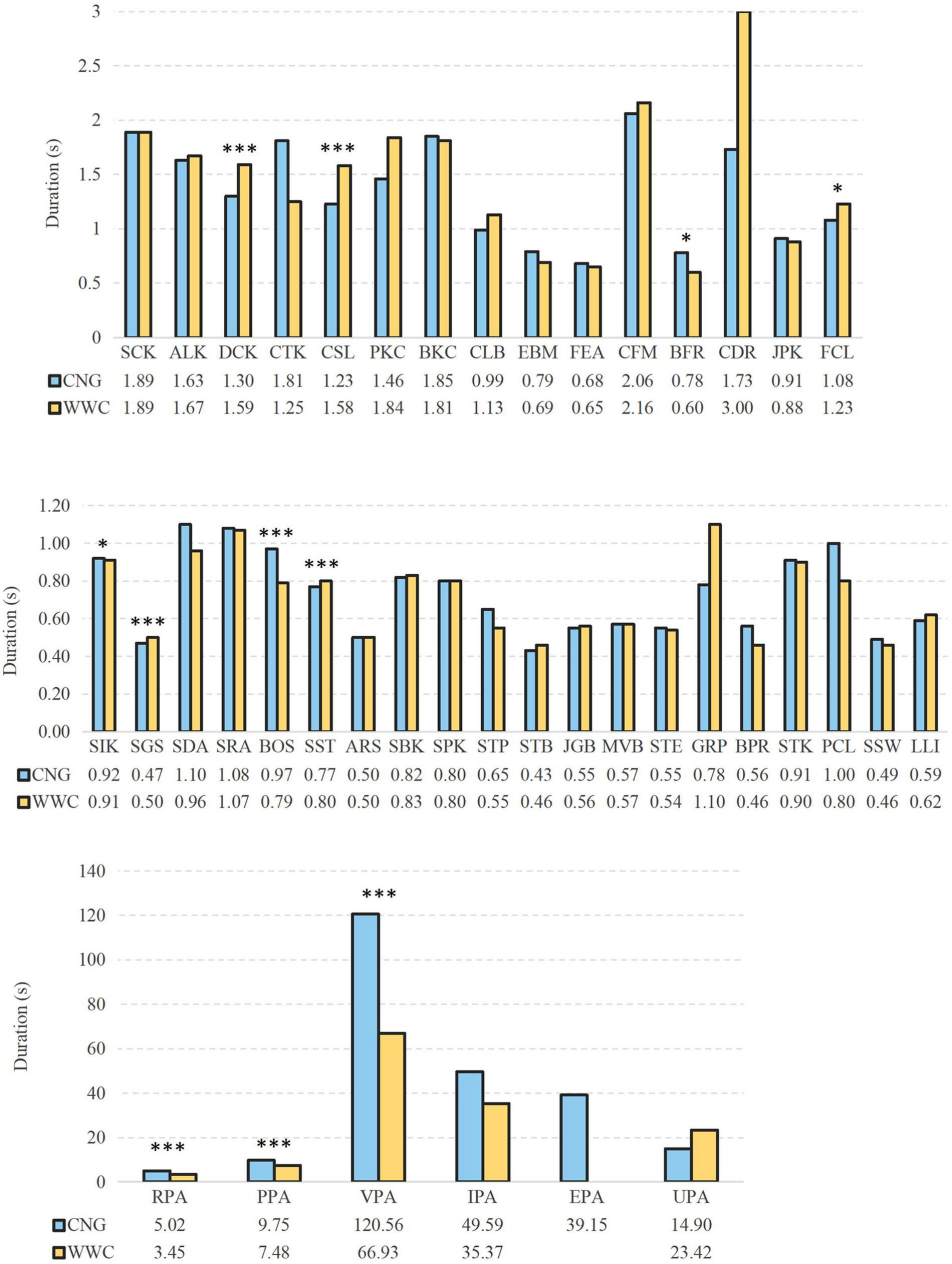
Durations of high-intensity, low-intensity and referee pause activities that differed significantly between tournaments.

Significant between-tournament differences were identified for five HIA indicators (Table 2, Figure 2). WWC showed longer durations in directional-change kicks ≥ 2 (*Z* = −7.111, *p* < .001, *r* = .54, large), consecutive slides ≥ 2 (*Z* = −14.794, *p* < .001, *r* = .80, very large), feinting actions (*Z* = −2.859, *p* = .008, *r* = .45, moderate, with *q* > .05), and forceful clinching (*Z* = −3.066, *p* = .002, *r* = .23, small), but shorter body rotation ≥360° (*t* = 3.103, *p* = .004, *d* = 0.49, small).

#### 3.2.3 Low-intensity activities

Among the 20 LIA indicators, only turning kick × 1 was normally distributed in both tournaments (Shapiro–Wilk *p* > .05); all others violated normality (all *p* < .05). Turning kick × 1 was compared via independent-samples t-test, and the remaining indicators via Mann–Whitney U tests.

Significant differences were observed for five LIA indicators (Table 2, Figure 2). WWC demonstrated shorter single kicks (*Z* = −2.794, *p* = .005, *r* = .21, small), bouncing steps (*Z* = −5.583, *p* < .001, *r* = .43, moderate), and stance switching (*Z* = −2.017, *p* = .007, *r* = .54, large, with *q* > .05), yet longer static standing (*Z* = −4.377, *p* < .001, *r* = .33, small) and single slides (*Z* = −5.584, *p* < .001, *r* = .43, moderate).

#### 3.2.4 Referee pauses

All six RP indicators violated normality (Shapiro–Wilk *p* < .05) and were analysed using Mann–Whitney U tests. WWC exhibited significantly shorter RP durations for regular pauses (*Z* = −10.105, *p* < .001, *r* = .77, very large), penalty pauses (*Z* = −6.412, *p* < .001, *r* = .49, moderate), and video-review pauses (*Z* = −4.187, *p* < .001, *r* = .32, small) compared with CNG (Table 2, Figure 2).

### 3.3 Intensity ratios

**Figure 3:**
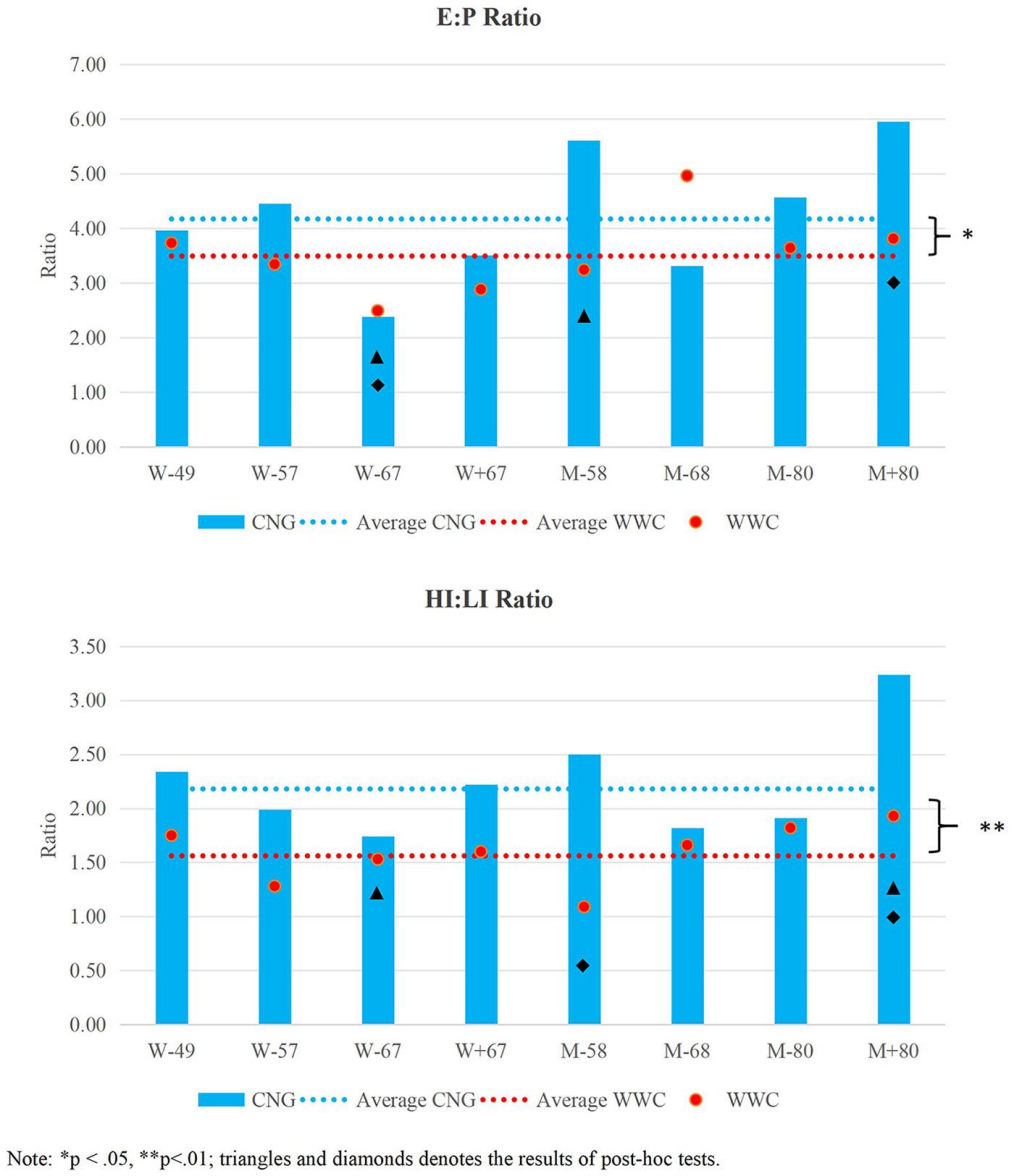
Intensity distributions across weight categories and overall intensity (Effort:Pause, high intensity activities: low intensity activities) by tournament.

ART ANOVA was applied following verification that E:P and HIA:LIA ratios violated normality assumptions (Shapiro–Wilk, all *p* < .001). For the E:P ratio, significant main effects of tournament [F(1, 122) = 3.273, *p* = .041, *η*^2^p = .001, very small] and weight class [F(7, 122) = 3.778, *p* = .001, *η*^2^p = .179, large] were observed, along with a significant Tournament × Weight Class interaction [F(7, 122) = 2.805, *p* = .010, *η*^2^p = .140, moderate]; whereas the main effect of round [F(2, 122) = .013, *p* = .0911, *η*^2^p = .051, small] and all interactions [Tournament × Round: F[2, 122] = .464, *p* = .630, *η*^2^p = .008, small; Round × Weight Class: F[14, 122] = .991, *p* = .466, *η*^2^p = .103, moderate; Tournament × Round × Weight Class: F[12, 122] = 1.146, *p* = .332, *η*^2^p = .094, moderate] were non-significant (Table 3, Figure 3). *post-hoc* pairwise comparisons for weight class revealed significant differences between weight classes 3 and 5 (*p* = .006) and between weight classes 3 and 8 (*p* < .001). The observed E:P ratio was 1:3.49 at WWC vs. 1:4.17 at CNG, indicating a denser activity profile at the World Championships.

**Table 3:** Three-way aligned rank transform (ART) ANOVA results for E:P and HIA:LIA ratios.

Ratio	Source	*df*	*F*	*p*	η^2^
E:P	Tournament (T)	1	3.273	.041*	.001
	Weight Class (W)	7	3.778	.001*	.179
	Round (R)	2	.013	.911	.051
	T × W	7	2.805	.010*	.140
	T × R	2	.464	.630	.008
	R × W	14	.991	.466	.103
	T × R × W	12	1.146	.332	.094
	Residual	122	—	—	—
Hi:LI	Tournament (T)	1	8.016	.005*	.061
	Weight Class (W)	7	3.328	.003*	.158
	Round (R)	2	.334	.717	.005
	T × W	7	3.156	.004*	.151
	T × R	2	1.366	.259	.022
	R × W	14	1.337	.195	.131
	T × R × W	12	.589	.848	.054
	Residual	124	—	—	—

* *p* < .05.

For the HIA:LIA ratio, significant main effects of tournament [F(1, 124) = 8.016, *p* = .005, *η*^2^p = .061, small] and weight class [F(7, 124) = 3.328, *p* = .003, *η*^2^p = .158, moderate] were observed, along with a significant Tournament × Weight Class interaction [F(7, 124) = 3.156, *p* = .004, *η*^2^p = .151, moderate]. The main effect of round [F(2, 124) = .334, *p* = .717, *η*^2^p = .005, trivial] and remaining interactions [Tournament × Round: F[2, 124] = 1.366, *p* = .259, *η*^2^p = .022, small; Round × Weight Class: F[14, 124] = 1.337, *p* = .195, *η*^2^p = .131, moderate; Tournament × Round × Weight Class: F[12, 124] = .589, *p* = .848, *η*^2^p = .054, small] were non-significant (Table 3, Figure 3). *post-hoc* pairwise comparisons for weight class revealed significant differences between weight classes 3 and 8 (*p* = .01) and between weight classes 5 and 8 (*p* = .018). The observed HIA:LIA ratio was 1:1.56 at WWC vs. 1:2.18 at CNG.

## 4 Discussion

The principal finding of this study is that the temporal disparity between national and international elite taekwondo competition is not a function of round duration, but rather of the density with which fight-relevant activities populate the available time. While macroscopic temporal boundaries were equivalent, World Championship (WWC) bouts exhibited longer high-intensity activity (HIA) durations, shorter low-intensity activity (LIA) durations, and more efficient referee pauses, yielding superior effort-to-pause (E:P) and high-to-low-intensity (HIA:LIA) ratios. These results carry important implications for how coaches should structure training load and technical development for athletes transitioning to international competition. However, this study merely results describe correlational patterns between two tournament systems; and does not establish that the temporal structure differences are either a cause or a consequence of competitive outcomes. The following sections interpret these findings while acknowledging that alternative explanations are equally consistent with the data.

### 4.1 Macro-temporal stability

The absence of differences in round duration between the CNG and the WWC aligns with previous observations that elite taekwondo is tightly governed by timing and rule structures that transcend event level (13). However, equivalence in macroscopic time should not be interpreted as equivalence in physiological demand. As Bridge et al. (13) have demonstrated, it is the proportion of time spent in high-intensity activity-not absolute bout length-that distinguishes competitive levels. Our data confirm that national and international contests occupy identical temporal envelopes, yet the density of activity packed within those envelopes differs markedly. This underscores a critical methodological principle: macroscopic bout duration and microscopic internal-load distribution are largely independent dimensions. Relying solely on round or match length to quantify competitive load may obscure substantial differences in physiological and tactical stress.

### 4.2 Micro-temporal intensification at the world championships

At the microscopic level, WWC bouts displayed a consolidated competitive phenotype characterized by denser work periods and abbreviated recovery windows. Specifically, the prolonged execution times for directional-change kicks (≥2) and consecutive sliding steps (≥2) indicate that international medallists engaged in more complex, multi-planar technical sequences. These actions require greater neuromuscular coordination and extend the duration of effective engagement (28, 29), thereby increasing the density of work intervals. An equally plausible alternative is that longer execution times reflect more cautious or less decisive attack transitions under higher defensive pressure, or that international athletes require more time to complete similar technical actions due to tighter defensive coverage.

From a metabolic inference, longer HIA combined with shorter LIA plausibly could equally imply higher metabolic efficiency among international athletes (i.e., the same temporal density at lower physiological cost) (30). With the compression of low-intensity activity, coupled with longer stationary standing durations (a transitional state), international athletes appear to employ a pacing strategy of brief, deliberate recovery followed by immediate re-engagement, rather than the more rhythmic oscillatory recovery pattern observed at the CNG. Alternatively, shorter bouncing steps could reflect higher anxiety or tighter defensive positioning that reduces preparatory movement. Equally, longer static standing may reflect hesitation, scanning for defensive vulnerabilities, or a lower tempo of initiative-taking under higher competitive pressure.

Tactically, the findings are equally informative. Longer forceful clinching durations at the WWC suggest a preference for assertive close-quarter management. Alternatively, longer clinch durations could indicate less efficient clinch resolution, or a tactical environment where both athletes are more willing to engage in prolonged close-quarters exchanges rather than rapidly disengaging. While shorter body rotation ≥360° durations indicate more efficient positional adjustment without the postural instability associated with large-amplitude rotation (31). Conversely, shorter rotation durations may reflect a tactical preference for linear over rotational attacks, or limited rotational technical capacity among international athletes who prioritise direct scoring angles over complex turning techniques. Shorter single-kick durations at WWC may indicate more efficient technical execution. However, they may also reflect less frequent use of single techniques as primary attacks, with international athletes preferring combination sequences that merge individual kicks into longer HIA bouts. Similarly, shorter single slides may indicate either more efficient footwork or less exploratory movement due to higher perceived risk. In contrast, athletes at CNG exhibited more frequent but shorter single sliding steps and longer stance switching, implying less decisive distance control and a greater reliance on low-intensity positional shuffling that may cede tactical initiative.

Del Vecchio et al. (20) compared regional- and state-level taekwondo competitions within Brazil and reported longer HIA durations and superior E:P ratios at the higher competitive level, consistent with our finding that WWC bouts exhibited longer HIA than CNG bouts. However, where Del Vecchio observed HIA differences of approximately 8% between levels, our observed HIA difference (with effect size *r* = .54) suggests a larger gap between national and international tournament systems than within a single competitive hierarchy. This discrepancy may reflect genuine differences between Chinese domestic and international competitive environments, or it may be attributable to the post-2022 rule intensification that was not present in Del Vecchio's pre-2016 data.

Comparison of our data with previous time-motion analyses (13, 16–20) reveals that both WWC and CNG bouts demonstrated substantially elevated competitive intensity relative to historical benchmarks. This elevation is likely attributable to the 2022 rule modification reducing the non-engagement penalty window from 10 s to 6 s (26), which compels athletes to initiate attacks more rapidly. Our results confirm that this rule change has successfully elevated global competitive intensity; however, the WWC maintains a higher temporal density than the CNG, indicating a persistent hierarchy of intensity even within this intensified landscape.

### 4.3 Referee pause dynamics

Referee pauses—encompassing regular, foul, and video-review stoppages—are among the most frequent interruptions in taekwondo competition (32). At the WWC, significantly shorter individual pause durations contributed to a faster overall competitive rhythm. It is important to contextualize this finding within the divergent video-review protocols: the WWC operates under World Taekwondo's “any head kick reviewable” regulation, whereas the CNG follows national federation rules prohibiting appeals against head-kick scoring. Paradoxically, despite a far greater absolute number of video reviews at the WWC (71 vs. 19), the mean duration per review was substantially shorter (66.93 s vs. 120.56 s). This likely reflects more intensive pre-tournament referee preparation, more streamlined video assistant referee (VAR) infrastructure, and stricter time-management protocols at the international level (33). The resulting reduction in dead time compresses the overall match cadence, forcing athletes to re-engage more rapidly and maintain cognitive arousal across shorter inter-bout intervals.

### 4.4 Intensity ratios and ecological validity

Both tournaments produced E:P and HIA:LIA ratios that exceeded previously reported taekwondo ranges (historically ∼1:2 to 1:8 for E:P and ∼1:3 to ∼1:7 for HIA:LIA) (19–25). The present WWC data (E:P = 1:3.49; HIA:LIA = 1:1.56) and CNG data (1:4.17 and 1:2.18) confirm that the most recent rule modifications have successfully elevated global competitive intensity (21, 22). Critically, even after excluding referee pause time (i.e., HIA:LIA ratio), the WWC exhibited a superior ratio, demonstrating that the intensity gap between international and national elite competition is not merely an artifact of pause-rule differences, but reflects genuinely denser movement patterns.

Additionally, we observed a notable intensity hierarchy across sex and weight classes, yet no significant between-round differences were detected. This finding aligns with Ouergui et al.'s observation that heavyweight competitors exhibit minimal round-to-round variation in activity intensity (18). Specifically, female middleweight athletes demonstrated denser activity profiles than both male lightweight and male heavyweight competitors in E:P ratio, whereas male heavyweights displayed the sparsest high-intensity engagement relative to low-intensity activity compared with female middleweights and male lightweights in HIA:LIA ratio. Slimani et al. previously reported lower intensity ratios among heavyweight competitors and superior ratios among male athletes (19); however, their data were derived exclusively from pre-2022 competitions. The present findings suggest that the current rule modifications have reversed these historical patterns: female athletes now exhibit intensified activity profiles, while heavyweight competitors demonstrate reduced relative high-intensity engagement, indicating a sex- and weight-specific temporal restructuring under the revised competitive framework.

For context, these values remain well below ratios reported for judo (∼2:1) (34), wrestling (∼2:1) (35), and Brazilian jiu-jitsu (∼6:1 to 9:1) (36), indicating that taekwondo retains its distinctive intermittent, kick-dominant character, yet is evidently evolving toward greater sustained engagement.

### 4.5 Consistency across competitive levels

Of the 41 indicators examined, 31 showed no significant between-tournament differences. This broad consistency has important interpretive implications. The absence of differences in the majority of indicators suggests that fundamental technical patterns—such as basic kicking frequencies, standard defensive movements, and routine referee stoppage categories—are largely preserved across national and international competition levels. The divergence is concentrated in specific tactical domains: multi-directional combination sequences (directional-change kicks ≥ 2), clinch management (clinch breaking, forceful clinching), footwork complexity (consecutive slides ≥ 2), and pause efficiency (regular, penalty, and video-review stoppages). This pattern implies that the temporal distinction between CNG and WWC is not one of comprehensive technical superiority but rather of selective tactical emphasis and competitive rhythm management. Coaches should therefore focus training adaptations on these specific domains rather than assuming a global intensity deficit.

### 4.6 Practical applications

These findings carry several evidence-based implications for elite taekwondo preparation, framed as hypotheses for training implementation rather than validated prescriptions:

#### 4.6.1 Training load reconstruction

Coaches should design training sessions that replicate the compressed low-intensity and extended high-intensity activity patterns observed at the international elite level. This may be achieved by prolonging high-intensity bout durations while shortening inter-bout recovery periods. Modified sparring protocols or small-sided games that restrict bouncing-step recovery and mandate rapid re-engagement may facilitate physiological adaptation to denser competitive schedules, though the transfer of such training adaptations to competitive performance requires empirical validation.

#### 4.6.2 Technical efficiency development

Emphasis should be placed on directional-change kicks, consecutive sliding entries, and combination sequences to extend effective attack windows. Concurrently, training should seek to minimise unproductive high-amplitude body rotation (≥360°) and passive clinching behaviours that do not contribute to scoring or tactical advantage.

#### 4.6.3 Tactical clinch proficiency

Given the dominant role of clinch breaking and forceful clinch durations at the world championship level relative to their underrepresentation at national-level competition, dedicated drill segments should be incorporated into preparatory programmes for athletes transitioning to international competition. However, the optimal duration and intent of clinch engagement (offensive vs. defensive) should be determined through tactical analysis rather than temporal metrics alone.

#### 4.6.4 Static-to-dynamic explosiveness

As stationary standing durations were longer at the world championship—suggesting a deliberate tactical pause before explosive engagement—athletes should practise initiating high-velocity kicks from static or near-static postures to replicate the transition demands of elite international competition. Whether longer static standing represents deliberate tactical preparation or momentary hesitation should be clarified through coach-athlete consultation and video-tactical analysis.

### 4.7 Limitations

This study is not without limitations. First, the cross-sectional comparative design cannot establish causality: temporal structure differences between CNG and WWC may reflect athlete preparation, officiating conventions, tactical cultures, or rule interpretation differences rather than competitive level. Second, although the 41-indicator taxonomy was Delphi-validated, it lacks biomechanical or physiological verification of metabolic intensity for each category; future research should integrate wearable technology (e.g., accelerometry, heart-rate telemetry) to anchor temporal classifications to physiological load. Third, the substitution of +73 kg and +87 kg categories for the absent +67 kg and +80 kg categories at the WWC, while justified by identical Chinese athlete rosters, introduces an ecological constraint: the international athlete composition in these substituted categories may differ from what would be expected in the original Olympic weight classes. Fourth, rule differences regarding video review may independently influence referee behaviour separate from athlete performance; however, the HIA:LIA ratio calculated excluding referee pauses confirms that the intensity gap is not merely an artifact of pause time. Fifth, the sample was restricted to gold-medal bouts, introducing selection bias: gold-medal athletes are the top performers at each event, and the temporal gap between overall tournament cohorts may be larger than the gap between gold-medalists, suggesting our effect sizes may underestimate the true intensity differential between domestic and international competition. Sixth, sex was not analysed as a stratification variable; men's and women's taekwondo bouts may differ in temporal structure, and future research should perform sex-stratified analyses or explicitly justify aggregation. Seventh, aggregating across all rounds and athletes masks individual strategic variation (attacker vs. defender, leading vs. trailing); exploratory athlete-level or score-margin stratification would strengthen future work. Finally, the performance of Chinese athletes at the WWC merits explicit acknowledgment. Among the Chinese contingent, one athlete advanced to a gold-medal bout but did not secure a medal, while the majority of remaining participants won bronze medals. This distribution suggests that Chinese athletes possess the competitive capacity to progress to late-stage rounds yet encounter challenges in medal conversion at the highest level. Consequently, the “maladaptation” narrative proposed in this study should be interpreted cautiously: it may reflect specific technical-tactical or psychological deficits in high-stakes medal bouts rather than systemic competitive inadequacy. Future research incorporating direct Chinese athlete data at WWC finals is needed to clarify this distinction.

## 5 Conclusions

This study demonstrates that the temporal distinction between national and international elite taekwondo competition resides not in macroscopic bout duration, which remains stable across event levels, but in the microscopic density of fight-relevant activity. World Championship bouts were characterised by longer high-intensity activity durations, compressed low-intensity recovery windows, and more efficient referee pauses than national-level competition, yielding superior effort-to-pause and high-to-low-intensity ratios. These findings reveal that international elite athletes employ more metabolically demanding movement patterns that may impose greater metabolic demands, pending direct physiological validation through wearable monitoring. The intensity disparity persists independent of video-review protocol differences, confirming that it reflects genuine competitive density gradients rather than officiating artifacts. Practically, these results underscore the necessity for training programmes to emphasise intensity reconstruction, multi-planar technical efficiency, and static-to-dynamic explosiveness when preparing athletes for international competition. Future investigations should incorporate physiological validation of time-motion categories and broaden the sampling frame beyond medal bouts to encompass full competitive cohorts.

## Data Availability

The original contributions presented in the study are included in the article/Supplementary Material, further inquiries can be directed to the corresponding author/s.
