# Supplementary material for: Temporal structure of national vs. world-level taekwondo competitions: a time-motion analysis in taekwondo
Source: Front Sports Act Living. 2026 Jul 22;8:1897013. doi: 10.3389/fspor.2026.1897013 (PMC13438380; doi:10.3389/fspor.2026.1897013)
Supplement: Supplementary file 1 [file Supplementaryfile1.docx]

**Supplementary Material: Composition and definition of high-intensity activities (HIA), low-intensity activities (LIA) and referee pause activities (RP)**

|  | **Type** | **Code** | **Definition** |
| --- | --- | --- | --- |
| HIA | Same-leg consecutive kicks ≥2 | SCK | Two scenarios: (a) the athlete executes two or more consecutive kicks without landing, striking the same target area or region each time; (b) the athlete completes one full kicking motion, lands without pause, and immediately executes a second kick, performing two or more repetitions. |
|  | Alternating-leg kicks ≥2 | ALK | The athlete performs complete kicking actions with alternating legs, landing after each kick and executing the next without pause, for two or more repetitions. |
|  | Directional-change kicks ≥2 | DCK | The athlete executes two or more consecutive kicks without landing, with each kick striking a different target area or region. |
|  | Consecutive turning kicks ≥2 | CTK | The athlete performs two consecutive turning kicks without pause. |
|  | Consecutive slides ≥2 | CSL | The athlete executes two or more simultaneous bilateral foot movements (both feet moving together), in forward or backward direction. |
|  | Push-and-kick combinations ≥2 | PKC | The athlete executes a pushing action while simultaneously performing a kick, for two or more repetitions. |
|  | Block-and-kick combinations ≥2 | BKC | The athlete executes a kick while simultaneously performing an obvious block to defend against the opponent's kick, for two or more repetitions. |
|  | Clinch breaking | CLB | The athlete uses the knee or leg to raise and disrupt the opponent's attacking action; also includes any kick executed after the breaking action without landing. |
|  | Evasive body movement | EBM | The athlete executes large-amplitude displacement away from their current position to evade the opponent's attack, typically accompanied by substantial head movement. |
|  | Feinting actions | FEA | Deceptive movements executed through body sway, coordinated hand or foot motions to mislead the opponent. |
|  | Consecutive footwork movements ≥2 | CFM | The athlete performs two or more footwork movements without pause, connecting to blocking, kicking, or other complete actions. |
|  | Body rotation ≥360° | BFR | The athlete rotates the body beyond 360° without executing any kicking technique. |
|  | Consecutive double roundhouse kicks ≥3 | CDR | The athlete executes three or more consecutive double roundhouse kicks (narae chagi). |
|  | Jumping kick | JPK | The athlete leaves the ground through aerial jumping, completing one or more kicks while airborne. |
|  | Forceful clinching | FCL | The athlete purposefully and actively applies forceful body contact against the opponent to disrupt close-range attacks or avoid stepping out of bounds. |
| LIA | Single kick | SIK | The athlete completes one full kicking action, with no subsequent high-intensity activity connected. |
|  | Single slide | SGS | The athlete executes one simultaneous bilateral foot slide (both feet moving together), with no subsequent high-intensity activity connected. |
|  | Short-range distance adjustment | SDA | The athlete uses a single foot to make small-range forward or backward movement to alter the distance from the opponent. |
|  | Short-range direction adjustment | SRA | The athlete uses a single foot to make small-range lateral movement to change facing direction relative to the opponent, typically to adjust boundary distance or follow the opponent's movement. |
|  | Bouncing step | BOS | The athlete performs slight vertical bouncing on the spot with both feet, heels leaving the ground while the balls of the feet remain in contact with the floor. |
|  | Static standing | SST | The athlete stands in place with no obvious foot movement, maintaining an almost stationary posture. |
|  | Advancing / retreating step | ARS | The athlete extends or withdraws one foot in any direction, covering greater range than short-range distance or direction adjustments. |
|  | Single block-and-kick | SBK | The athlete executes one kick while simultaneously performing an obvious block to defend against the opponent's kick, with no subsequent high-intensity activity connected. |
|  | Single push-and-kick | SPK | The athlete executes a pushing action while simultaneously performing one kick, with no subsequent high-intensity activity connected. |
|  | Straight punch | STP | The athlete strikes the opponent's trunk with a straight punch (jireugi). |
|  | Stationary blocking | STB | The athlete stands in place, using the upper limbs to block the opponent's attack. |
|  | Jumping / switching guard block | JGB | The athlete uses jumping to change height, or switches guard to alter body orientation, to obstruct the opponent from striking valid scoring areas. |
|  | Moving block | MVB | The athlete moves forward or backward to change distance while simultaneously using the upper limbs to block the opponent's attack. |
|  | Stationary evasion | STE | The athlete remains in position without leaving their current location, using minimal movement to evade the opponent's attack, typically accompanied only by slight head movement. |
|  | Grabbing / pushing | GRP | The athlete grabs or pushes the opponent without connecting to any leg technique; identified after referee penalty call. |
|  | Body rotation < 360° | BPR | The athlete rotates the body less than 360°, without executing any kicking technique. |
|  | Single turning kick | STK | The athlete executes one turning kick (dollyo chagi). |
|  | Passive clinching | PCL | Natural body contact between the athlete and opponent, with neither party applying active force. |
|  | Stance switching | SSW | The athlete performs a single conversion of body orientation without contact with the opponent. |
|  | Leg lifting | LLI | The athlete raises the leg without executing a kick, allowing it to lower naturally. |
| RP | Regular pause | RPA | Includes mutual clinching without offense, non-penalty falls, protective equipment dislodgement, or similar situations; no penalty is issued following the pause. |
|  | Penalty pause | PPA | A pause occurring after a foul action; the referee identifies and penalizes the foul following the stoppage. |
|  | Video review pause | VPA | A pause initiated by coach appeal or self-initiated video review by the head referee, involving video replay review. |
|  | Injury pause | IPA | A pause occurring when an athlete is injured, identifiable by medical staff entering the competition area following the stoppage. |
|  | Equipment malfunction pause | EPA | A pause caused by electronic equipment malfunction or related technical failures. |
|  | Unexpected incident pause | UPA | A pause caused by unanticipated circumstances that disrupt the normal progression of the match. |

Note: HIA: Onset-the instant either foot left the ground or the upper limb initiated an attacking action; Offset-the moment (1) the limb that executed the final kick or punch returned to the ground or a defensive position, (2) the limb used for punching or blocking retracted to the ready posture, (3) the technical action caused the opponent to fall, or (4) the referee signaled a stoppage.

LIA: Onset-the athlete's disengagement from the confrontation in the absence of other pause conditions; Offset-the moment (1) the athlete re-entered HIA or (2) the referee issued the "Kal-yeo" command and gesture.

RP: Onset-the referee's issuance of the "Kal-yeo" command and gesture, the athlete falling, or the occurrence of a non-combat pause (e.g., medical care, video review, equipment malfunction); Offset- the referee's issuance of the "Kye-sok" command and gesture, at which point the match resumed.
